# French Requirements and the D.D.S. in France

**Published:** 1905-01-15

**Authors:** Chas. J. Gray

**Affiliations:** Petoskey, Mich., President Michigan Dental Board of Examiners


### FRENCH REQUIREMENTS AND THE D.D.S. IN FRANCE.

CHAS. J. GRAY, D.D.S., PETOSKEY, MICH.
President Michigan Dental Board of Examiners.

The American practitioners in France are, as a rule, men enjoying large and lucrative practices. They are respected by the American colony and the intelligent part of the French population. They are disliked by the French practitioners of dentistry, deserted by the American Colleges which graduated them, and allowed to individually work out their own salvation. It must be pleasant for these legitimate and ethical practitioners to see certain colleges under the sway of the National Association of Dental Faculties, turn out yearly as full-fledged Doctors of Dental Surgery the crowd, from Europe, who annually are admitted illegally into our colleges. Despite rules to the contrary some colleges admit students with little knowledge of the English language, and proceed to matriculate them, and teach them English and graduate them in seven months!!

My sympathies go out to my American brethren practicing in Europe, who are subjected to such indignities at the hands of their own countrymen. It is the meanest thing the colleges do. I am glad that the men now practicing in France have become so hardened that they can endure it, but I frankly confess that I could not settle down among foreigners and be happy when, at every turn, the "D.D.S." was assailed by the French dentists and the press. Remarks such as follow, are heard everywhere. "It can be bought." "Very easily obtained." "Just six months' attendance is all that is required to get it." "Men have left France, unable to talk a word of English and have returned in six months, still unable to make themselves understood in English and yet bring back an American diploma." Quoting from a French Dental Journal:

"Un etranger, arme d'un diplome plus ou moins douteux, n'ayant aucune valeur en France, obtenu en Angleterre ou en Amerique, le plus souvent sine curriculum, arrive en France y joue le grand personnage, etc., etc."

(A foreigner, armed with a diploma more or less doubtful, having no value in France, obtained in England or in America most often without curriculum, arrives in France and plays the part of a great personage, etc. etc.)

Such remarks or analogus ones, are heard constantly on all sides, with the result that they very frequently find credence in the minds of otherwise very intelligent people. That these remarks are hypocritical can easily be proved by one who has lived there and knows their ways. A walk along the main boulevards of Paris will disclose the fact that a quarter of the hand-bills given out are referring to dentistry. They all exploit American dentistry and invariably wind up by announcing that a person possessing an American degree is in attendance. So that you see, "deep down," the American dental degree is respected and they all know that the mere fact of them having spent a few months in any of our colleges is a valuable acquisition to them before the public,. From a personal knowledge of affairs in Europe, I will say that there are Frenchmen in France, possessed of the American degree in dentistry, who never had any preliminary education and who cannot talk a dozen words of English. In nearly all cases these degrees have been granted by two or three Eastern Colleges, and they should be subjected to heavy punishment from the N. A. D. F. If that fails, the State Societies should seek redress from their legislatures. It is certainly wrong to send those men back and place them on a par with the legitimate owner of the degree.

Just now all the colleges are deeming it big to have foreigners in their classes, and it is about time that a halt was called upon them, for they are simply damning the American degree abroad. Give the man, coming to us well-equipped, all the chance in the world, but don't accept any foreigner who is willing to stay only one session.

I will briefly outline educational conditions in France, and the reader can make his own comparisons. Frequent quotations will be made from data furnished me while in Paris, in October last, by my friend Dr. Geo. A. Roussell, a member of the advisory board for France of the Foreign Relations Committee.

There are at present existing in France five dental schools—three in Paris, one in Lyons, one in Bordeaux—which are semi-officially recognized by the Government. In order to matriculate in any of these schools, a preliminary examination has to be passed before the Sorbonne (University of Paris). This examination is indispensable and no foreign equivalents are accepted in place of it. Of the three dental schools in Paris the "Ecole Dentaire de Paris" is the best equipped and the most progressive. Next to this comes the "Ecole Odontotechnique." This ranks but little inferior to the first. It is more under the domain and conservative influence of strictly medical men and is inferior to its rival in the completeness of teaching in all the practical branches. The third school the "Ecole Dentaire Francaise," should not be taken seriously at all. It is merely recognized as giving the necessary courses required for the examination before the Faculty of Medicine.

Candidates for the degree of Surgeon Dentist must present one of the three following papers before they can matriculate in any of the dental schools:

1. Diploma of Bachelor.
2. Certificat d' Etudes Primaires Superieures.
3. Certificat d' Etudes.

Of these, the third is the one usually chosen by the student. The examination for this certificate is given by the Rector of the Sorbonne after an examination before a jury made up of an inspector of the academy and three Professors Agreges in the departments of classic or modern instruction. The examination is divided into two parts, and only those who are successful in the first or written are allowed to take the second or oral. The written examination consists of a composition in French of about 800 to 1,200 words on a subject announced at the time of the test, and three hours is allowed for it. A translation into French from a foreign language is also required. This consists of the rendering into correct French of a selection of about 200 words, taken from a standard author in either Latin, German, English, Italian or Spanish.

The oral examination embraces French literature and grammar, and the elements of arithmetic, algebra, geometry, physics, chemistry, geology, zoology and botany.

The French Government places special stress upon the preliminary examination of persons desiring to practice dentistry. It has made known the fact that in the near future it will require of dental and medical students alike, that they possess the degree of Bachelor as a foundation for their studies. Their determination is to make their professional men, men of broader education, capable not only of speaking and writing correctly their mother tongue, but men who shall be familiar with their classic and modern literature and well grounded in the principles fundamental to a higher education. After passing one of the three examinations mentioned above, the student is allowed to enter one of the dental schools and takes what is called his first "inscription." The school course lasts three years of nine months each, and every three months an additional "inscription" is taken. These "inscriptions" are equally good in all schools and the student may change if he so desires.

When twelve "inscriptions" have been taken, the candidate is allowed to come before the Medical Faculty of the University of France for final examination. This Faculty alone confers the right to practice dentistry, and with it, the degree of Surgeon Dentist.

The student may or may not, at wish, have passed the final examinations at his school, which gives a diploma of no legal value, and which would pass as a doubtful honor should the student fail later before the Faculty of Medicine.

The examinations before the Medical Faculty are held either at the medical school or at one of the hospitals, equipped with dental infirmary and clinic. Each examination is conducted by a jury of three professors of the Medical Faculty, except the third, in which two dentists form part of the jury. The candidate is examined in turn by each of the three examiners. The examining board sits twice a year and the three examinations of each session are held at intervals of one month.

Foreigners who have obtained diplomas abroad and who wish to practice in France, are obliged to pass the same examinations as the French students, beginning with the entrance examination. However, the law allows a partial or total dispensation of school work, if a request for the same meets with the approval of an advisory board, which passes upon the credentials presented. Up to the present time a dispensation of two years' school attendance has been granted in three cases only. Following are the three examinations before the Medical Faculty given in general outline:

First Examination:—

1. General Anatomy and Physiology (very thorough).
2. Special Anatomy and Physiology of the Mouth (very thorough).

Second Examination:

1. General Pathology and Therapeutics (very general).
2. Special Pathology and Therapeutics of the Mouth (very general).
3. Anesthetics and Antiseptics (very general).

Third Examination:—

1. Prosthetic and Operative Dentistry (easy).

This third examination comprises an oral examination, principally on diseases of the mouth and adjoining regions, and on prosthetic and operative dentistry, but may include any of the subjects of the first two examinations. The candidate is given a patient for examination and is required to note on a chart all abnormalities of the teeth and mouth, and if necessary, proceed to full examination of patient by questions and physical examination. The student may be required to perform some operation such as extraction, use of cautery, lancing abscess, but this is about the limit of the practical test.

As will be seen, the examination before the Faculty of Medicine is a rather severe and difficult one, inasmuch as the questions asked are apt to be very general and appertain in many instances to the domain of medicine and surgery. There is unfortunately no very severe or complete technical or practical test attempted by the Faculty of Medicine. They seem to consider the theoretical and scientific aspects of the examination for the diploma of Chirurgien Dentiste the more necessary, consequently, the students who are given this diploma often possess but meager qualifications for the every-day's practical operations, which the dentist is called upon to perform.

The dental schools of course are more or less hampered in the benefits which they might confer upon our profession by this fact, and it cannot be denied that the students give more serious attention to theoretical and scientific branches of study than to the practical, and the schools are more or less obliged to recognize the former at the expense of the latter. The present law in France confers upon all French Doctors of Medicine the *right,* without any supplementary studies or training, to practice dentistry, and indeed the tendency in France is undoubtedly towards the swallowing up of the dental by the medical profession. The French schools are not endowed or State institutions and are consequently dependent on the fees of their patients for support. The average infirmary patient there is unable to pay anything like the required fee for the best class of work, consequently the French student is largely restricted to cheap operations, and although he may be theoretically well educated he graduates oftentimes poorly experienced in practical work of the best kind. The atmosphere of the French school, and the influence of the ordinary work is such that, on a three-year basis, the French graduate should in my opinion be given credit only for the work that he can "show good" in. This would properly give him one year's credit and excuse him in some of the theory work of the second and third year, but he should, in all cases, be compelled to take and pass our preliminary examination in English before being given any credit at all.

As it is, the D.D.S. wishing to obtain the French diploma of Chirurgien Dentiste is obliged to spend from one to two years in studying the French language and in preparing for and passing the entrance examinations in French, even though he may be the holder of a degree in arts or sciences such as B.S., B.A., or M.A. He then is required to spend two years in a French dental school before he can take the examination for the French dental diploma. Yet, a French graduate wishing to obtain the American diploma of D.D.S., can do so in two years without preliminary examinations. The comparison is not favorable to the value or honor of the American degree in European eyes. To further "rub it in" the special dispensation clause has been altered so that Americans must now take two full years in French dental schools. Some of our colleges have been known to boast of their monopoly of foreigners. Their deans have gone so far as to put advertisements in the British and French daily papers stating, that Dr. "Blank" would be at hotel———London or Paris during certain hours of certain days for the purpose of interviewing prospective students for their institutions. No wonder then that the terms used in the first part of this paper are often expressed. The French themselves have established the precedent as far as these foreign equivalents are concerned, and if the American colleges were true to themselves and their graduates they, too, would set a standard and abide by it. By so doing they would give the best answer possible to the damning reports of the French Medical and Dental press. In conclusion, I believe that it is the duty of our National Association of Dental Faculties to so legislate that the American diploma should be made to stand for just what it is—the reward for work well and truly done—whether by American or Frenchmen. Don't make the mistake that some are now making and pander to the "unknown." What has been said of France is true of all European countries only to a greater extent. The work in the British Isles is of a higher standard and requires more generous consideration at our hands.

